# The Banana Fruit SINA Ubiquitin Ligase MaSINA1 Regulates the Stability of MaICE1 to be Negatively Involved in Cold Stress Response

**DOI:** 10.3389/fpls.2017.00995

**Published:** 2017-06-12

**Authors:** Zhong-Qi Fan, Jian-Ye Chen, Jian-Fei Kuang, Wang-Jin Lu, Wei Shan

**Affiliations:** State Key Laboratory for Conservation and Utilization of Subtropical Agro-Bioresources/Guangdong Provincial Key Laboratory of Post-harvest Science of Fruits and Vegetables, College of Horticulture, South China Agricultural UniversityGuangzhou, China

**Keywords:** banana fruit, cold stress, E3 ligase, ICE1, ubiquitination

## Abstract

The regulation of ICE1 protein stability is important to ensure effective cold stress response, and is extensively studied in *Arabidopsis*. Currently, how ICE1 stability in fruits under cold stress is controlled remains largely unknown. Here, we reported the possible involvement of a SEVEN IN ABSENTIA (SINA) ubiquitin ligase MaSINA1 from banana fruit in affecting MaICE1 stability. MaSINA1 was identified based on a yeast two-hybrid screening using MaICE1 as bait. Further yeast two-hybrid, pull-down, bimolecular fluorescence complementation (BiFC) and co-immunoprecipitation (CoIP) assays confirmed that MaSINA1 interacted with MaICE1. The expression of *MaSINA1* was repressed by cold stress. Subcellular localization analysis in tobacco leaves showed that MaSINA1 was localized predominantly in the nucleus. *In vitro* ubiquitination assay showed that MaSINA1 possessed E3 ubiquitin ligase activity. More importantly, *in vitro* and semi-*in vivo* experiments indicated that MaSINA1 can ubiquitinate MaICE1 for the 26S proteasome-dependent degradation, and therefore suppressed the transcriptional activation of MaICE1 to MaNAC1, an important regulator of cold stress response of banana fruit. Collectively, our data reveal a mechanism in banana fruit for control of the stability of ICE1 and for the negative regulation of cold stress response by a SINA E3 ligase via the ubiquitin proteasome system.

## Introduction

Banana (*Musa acuminata*) is one of the most popular fruit crops worldwide (Paul et al., 2016; Shan et al., 2016). As a typical climacteric fruit, bananas have a very limited shelf-life due to rapid softening (Han et al., 2016). Practically, low temperature storage is an effective technology to maintain post-harvest banana fruit qualities and to extend its shelf-life. However, being tropical fruits, banana fruit are highly sensitive to cold stress, and storage at low temperatures (<13°C) results generally in peel browning and failure of ripening, causing severe post-harvest losses (Chen et al., 2008), which restricts the application of low temperature storage during transportation of banana fruit.

Cold stress is one of the major abiotic stresses limiting plant growth and development, productivity, product quality, post-harvest life and geographic distribution (Knight and Knight, 2012; Shan et al., 2014; Janmohammadi et al., 2015). To overcome this constraint, plants have developed sophisticated responses at the physiological and biochemical levels (Hu et al., 2013; Huang et al., 2015; Jia et al., 2016). Over the last decades, enormous progress has been achieved in the identification of important components involving in the cold signaling network, among which the INDUCER OF CBF EXPRESSION (ICE)–CREPEAT BINDING FACTOR/DRE BINDING FACTOR–COLD REGULATED (ICE-CBF-COR) transcriptional cascade is pretty well understood (Medina et al., 2011; Zhou et al., 2011; Wisniewski et al., 2014; Shi et al., 2015). In this pathway, *CBFs* are induced rapidly by cold stress, and in turn activate downstream *COR* genes to increase plant cold tolerance. ICEs encode MYC-type bHLH transcription factors (TFs) that can activate *CBFs* gene expression via binding to their promoters (Chinnusamy et al., 2003; Shi et al., 2015).

It is well-known that ICE-CBF-COR pathway is positively or negatively controlled by many important regulators at transcriptional, post-transcriptional, and post-translational levels. Among these regulators, CAMTA3 (calmodulin-binding transcription activator 3) (Doherty et al., 2009), SIZ1 (for SAP and Miz1) (Miura et al., 2007) and OST1 (OPEN STOMATA 1) (Ding et al., 2015) are positive regulators, while MYB15 (Agarwal et al., 2006), HOS1 (HIGH EXPRESSION OF OSMOTICALLY RESPONSIVE GENES1) (Lee et al., 2001; Dong et al., 2006; Jung et al., 2014) EIN3 (ethylene insensitive 3) (Shi et al., 2012) and JA ZIM-domain 1/4 (JAZ1/4) (Hu et al., 2013) function as negative regulators of ICE-CBF-COR pathway. For example, HOS1 ubiquitinates and degrades ICE1 protein via the 26S proteasome pathway, indicating that HOS1 attenuates cold responses by triggering ICE1 degradation through the ubiquitin-proteasome system (UPS) (Lee et al., 2001; Dong et al., 2006; Jung et al., 2014). On the contrary, a small ubiquitin-related modifier (SUMO) E3 ligase, SIZ1 sumoylates ICE1, antagonizing the polyubiquitination of ICE1 to facilitate its stability, thus causes enhanced cold tolerance (Miura et al., 2007). More recently, the protein kinase OST1 is also shown to phosphorylate ICE1 to enhance its stability and transcriptional activity, resulting in increased cold tolerance (Ding et al., 2015). These findings suggest that the regulation of ICE1 protein stability is important to ensure effective cold stress response. Although the UPS-mediated protein degradation is an important post-translational regulatory mechanism for controlling the abundance of key regulators, and has emerged as an integral player in plant response and adaptation to environmental stresses, its involvement in regulating ICE1 stability in relation to cold stress response of economical fruits, such as bananas, needs to be investigated.

Giving the increasing demand of cold storage and the cold sensitivity of banana fruit, we are aiming at the molecular mechanism(s) of the cold response in banana fruit, which will contributes to genetic improving cold tolerance, fruit quality and storage potential. Our previous studies have shown that two banana fruit MYC2 proteins act together with ICE1, which is related to the methyl jasmonate (MeJA)-induced chilling tolerance (Zhao et al., 2013). In addition, a cold-responsive NAC (NAM, ATAF1/2, and CUC2) TF MaNAC1, is a novel direct target of MaICE1 and may be associated with cold stress through interacting with MaCBF1 (Shan et al., 2014). Nevertheless, the factors controlling ICE1 protein stability associated with cold stress response of banana fruit are far from being clearly elucidated. In this study, we report that a SEVEN IN ABSENTIA (SINA) E3 ligase MaSINA1 interacts with and ubiquitinates MaICE1, leading to the degradation of MaICE1 and the attenuation of its transcriptional activity. Our study thus reveals that MaSINA1 may negatively regulate cold stress response of banana fruit via controlling MaICE1 stability.

## Materials and Methods

### Plant Materials and Treatments

Pre-climacteric banana (*M. acuminata*, AAA group, cv. Cavendish) fruit at 75–80% maturation (about 12 weeks after anthesis) were harvested from a local commercial plantation near Guangzhou, China. Each banana hand was cut into individual fingers. Banana fruit of uniform weight, shape and maturity, and free of visual defects, were used for this study. For cold stress, fruit were stored immediately at 7°C for 5 days, while for control, fruit were directly stored at 22°C. Samples were taken at 0, 6, and 12 h and 1, 3, and 5 days of storage. Banana peel was collected, frozen in liquid nitrogen and stored at -80°C for further use.

Tobacco (*Nicotiana benthamiana*) plants were planted in a growth chamber of 22°C under long day conditions (16-h light/8-h dark), and 4- to 6-week-old plants were selected for analysis.

### Yeast Two-Hybrid (Y2H) Screening/Assay

Yeast two-hybrid screening/assay was performed using the Matchmaker^TM^ Gold yeast two-hybrid system (Clontech, Cat. No. 630489) following the User Manual. Briefly, to screen the interacting proteins, the coding sequence of *MaICE1* was cloned into pGBKT7 vector to fuse with the DNA-binding domain (DBD) as bait, and transformed into yeast strain Gold Y2H by the lithium acetate method. The cDNA library (2.0 × 10^9^ cfu/ml) was generated by TAKARA BIOTECHNOLOGY (DALIAN) CO., LTD using poly (A)^+^ mRNAs extracted from banana fruit that were stored under cold stress, fusing to pGADT7 with activation domain (AD) and was transformed into Gold Y2H carrying the MaICE1 bait. The transformed cells (approximately 6.0 × 10^6^ cfu) were placed on DDO medium (minimal media double dropouts, SD medium with -Leu/-Trp), and positive clones among the transformants were identified by scoring growth on QDO medium (minimal media quadruple dropouts, SD medium with -Leu/-Trp/-Ade/-His). Plasmids of positive clones was extracted from the yeast cells using a TIANprep yeast plasmid DNA kit (Tiangen) and then transformed into *Escherichia coli* for sequencing.

To confirm the MaSINA1-MaICE1 interaction, the coding sequences of *MaSINA1* and *MaICE1* were inserted into pGBKT7 or pGADT7 vector as bait and prey, respectively, and were co-transformed into Gold Y2H. Yeast cells were grown on DDO medium for 3 days, then transformed colonies were plated onto QDO medium, as well as QDO media containing 4 mg mL-1 X-α-Gal (α-Gal) for blue color development, to verify the possible interaction between MaSINA1 and MaICE1 according to their growth status and the activity of α-galactosidase. Primers used for Y2H assay are listed in Supplementary Table 1.

### Bimolecular Fluorescence Complementation (BiFC) Analysis

To create constructs for BiFC assay, the coding sequence of MaICE1 or MaSINA1 fusing with YNE or YCE, was cloned into the pEAQ-HT vector (Sainsbury et al., 2009). The resulting constructs were then introduced into the *Agrobacterium tumefaciens* strain GV3101, and co-infiltrated into the abaxial side of 4- to 6-week-old tobacco (*N. benthamiana*) leaves using a 1-mL needleless syringe as described previously (Fan et al., 2016). Infected tissues were analyzed at 48 h after infiltration. YFP fluorescence was captured using the Confocal Spectral Microscope Imaging System (Leica TCS SP5), with an argon blue laser at 488 nm, a beam splitter for excitation at 500 nm, and a spectral detector set between 515 and 540 nm. Primers used for generating the constructs are listed in Supplementary Table 1.

### *In Vitro* GST Pull-Down Assay

The full-length cDNA of *MaSINA1* was cloned into the pMAL-c2X expression vector (New England Biolabs) (primers are listed in Supplementary Table 1). The maltose binding protein (MBP)-tagged MaSINA1 fusion protein was expressed in *BM* Rosetta (DE3) and purified by affinity chromatography using amylose resin (New England Biolabs, Cat. No. E8021S) according to the manufacturer’s instructions. GST-MaICE1 protein was obtained as described previously (Shan et al., 2014). *In vitro* GST pull-down assay was performed as described previously with minor modifications (Liu et al., 2013). Briefly, GST or GST-MaICE1 recombinant protein was incubated with 30 μL of Glutathione resin in 1× PBS buffer for 2 h at 4°C, the binding reaction was washed three times with 1× PBS buffer and then the MBP-MaSINA1 recombinant protein was added and incubated for an additional 2 h at 4°C. The beads were washed five times with wash buffer (1× PBS, 0.1% Triton X-100), following the elution by boiling with SDS loading buffer, separated by 10% SDS-PAGE, and subjected to western blotting analysis using the anti-MBP antibody (Abcam, Cat. No. ab9084) and the anti-GST antibody (Abcam, Cat. No. ab9085) respectively, with secondary goat anti-rabbit IgG peroxidase antibody (Thermo Scientific, Cat. No. 32460). Detection was carried out using the chemiluminescent substrate SuperSignal West Pico (Thermo Scientific, Cat. No. 34080) for horse-radish peroxidase and imaged on a ChemiDoc^TM^ MP Imaging System (Bio-Rad Laboratories).

### Semi-*In Vivo* Coimmunoprecipitation (CoIP) and Ubiquitination Assays

To create MaSINA1-His and MaICE1-GFP constructs, full-length *MaSINA1* or *MaICE1* was inserted into pEAQ-HT-His and pEAQ-HT-GFP vectors (primers are listed in Supplementary Table 1), respectively (Sainsbury et al., 2009; Peyret and Lomonossoff, 2013), and were introduced into *A. tumefaciens* strain GV3101, following co-infiltrated into the abaxial side of 4- to 6-wk-old tobacco leaves using a 1-mL needleless syringe. After 36 h of infiltration, 10 μM MG132 (Merck, Cat. No. 474790) was injected into tobacco leaves to prevent protein degradation. After 36 h, tobacco leaves were harvested and the protein was extracted as described by Han et al. (2016) and Ye et al. (2016), as well as the following CoIP assay. A 10 μl volume of anti-GFP antibody (Abcam, Cat. No. ab290) was added to 1 mL of cell lysates. Then, binding was gently shaked at 4°C for 4 h, and 50 μl of protein A agarose beads (Roche, Cat. No. 11134515001) was added. After 3 h of incubation at 4°C, the precipitated samples were washed, separated by SDS-PAGE and then performed western blotting analysis as described above using 4000-fold diluted anti-His antibody (Abcam, Cat. No. ab9108) and anti-GFP antibody (Abcam, Cat. No. ab290) respectively, for CoIP assay, and anti-ubiquitin antibody (Sigma–Aldrich, Cat. No. U119) for examining the ubiquitination of MaICE1.

### Gene Isolation, Sequence and Expression Analysis

Frozen banana peel was ground in liquid nitrogen using a mortar and pestle. Total RNA was extracted using the hot borate method of Wan and Wilkins (1994), and the extract was treated with DNAse I digestion using an RNAse-free kit (Promega, Cat. No. M6101). The DNA-free total RNA was used as template for reverse-transcription PCR. The first-strand cDNA of the product was applied to PCR amplification. Quantitative real-time PCR (qRT-PCR) was carried out on a Bio-Rad CFX96 Real-Time PCR System using the GoTaq^®^ qPCR Master Mix Kit (Promega, Cat. No. A600A) following the manufacturer’s instructions. *MaACT1* was used as the reference gene to normalize the gene expression levels (Chen et al., 2011). Primers for gene isolation and qRT-PCR are listed in Supplementary Table 1.

Alignments were carried out on ClustalX (version 1.83) and GeneDoc software, and a phylogenetic tree was constructed using the Neighbor-Joining method in the MEGA5 program.

### Subcellular Localization Assay

The complete Open Reading Frame (ORF) of *MaSINA1* was amplified and inserted into the pEAQ-GFP vector (primers are listed in Supplementary Table 1). The MaSINA1-GFP plasmid was electroporated into the *A. tumefaciens* strain GV3101, and injected into the abaxial side of 4- to 6-week-old tobacco leaves as described above. pEAQ-GFP was employed as the positive control. After 48 h of infiltration, GFP signal was visualized with a fluorescence microscope (Zeiss Axioskop 2 Plus) with a beam splitter for excitation at 500 nm.

### Promoter Isolation and Activity Analysis

Genomic DNA of banana leaves was extracted using the DNeasy Plant Mini Kit (Qiagen, Cat. No. 69104). The *MaSINA1* promoter region was amplified by PCR using the specific primers listed in Supplementary Table 1. Conserved *cis*-element motifs in the promoter were predicted using Plant-CARE^1^ database. The PCR product was inserted into the pGreenII 0800-LUC double reporter vector (Hellens et al., 2005) to fuse it with the Firefly luciferase (LUC) reporter gene (*MaSINA1* pro-LUC). A Renilla luciferase (REN) droved by the 35S promoter at the same vector was used as an internal control. The construct CaMV35S-REN/*MaSINA1* pro-LUC was infiltrated into tobacco leaf protoplasts by polyethylene glycol (PEG) methods as described previously (Shan et al., 2014; Fan et al., 2016).

The promoter activity was assayed according to Fan et al. (2016). The transformed protoplasts were incubated at room temperature (22°C) or cold (7°C). After 36 h, LUC and REN activities were measured on the Luminoskan Ascent Microplate Luminometer (Thermo) using the dual luciferase assay kits (Promega, Cat. No. E1910), with a 5-s delay and 15-s integrated measurements. The promoter activity is indicated by LUC/REN ratio. At least six assay repeats were included for each.

### *In Vitro* Ubiquitination Assay

The ubiquitination assay was generally conducted as described by Cheng et al. (2012). For E3 ubiquitin ligase activity assay of MaSINA1, 500 ng MBP-MaSINA1 recombinant protein was incubated for 2 h in the presence or absence of 50 ng of human E1 (Boston Biochem, Cat. No. E305), 250 ng of human E2 (Boston Biochem, Cat. No. E2-622), 2 mg of ubiquitin (Boston Biochem, Cat. No. u–100sc). The reaction products were subjected to western blotting using anti-MBP (Abcam, Cat. No. ab9084) and anti-Ub antibodies (Sigma–Aldrich, Cat. No. U119). For the *in vitro* MaSINA1-mediated MaICE1 ubiquitination assay, MBP-MaSINA1 and GST-MaICE1 proteins were coincubated as described above. The reaction products were analyzed using the anti-GST, -MBP and -Ub antibody, respectively.

### Semi-*In Vivo* Cell-Free Proteasomal Degradation Assay

To construct pEAQ-MaICE1-GFP-LUC, firstly the coding region of firefly LUC was amplified from pGreenII 0800-LUC as template, and inserted into pEAQ-GFP, generating pEAQ-LUC-GFP. Then the full length of *MaICE1* was cloned into pEAQ-LUC-GFP to fuse in frame with LUC and GFP to give rise to pEAQ-MaICE1-LUC-GFP. The full-length cDNA of MaSINA1 without the stop codon was cloned into pEAQ vector, generating pEAQ-MaSINA1. The specific primers used for construction these vectors are shown in Supplementary Table 1. The constructs were electroporated into the *A. tumefaciens* strain GV3101, growing in YEP medium (1% peptone, 1% yeast extract, and 0.5% NaCl) supplemented with 100 mg/L kanamycin and 10 mg/L rifampin overnight at 28°C. Cells were centrifuged, resuspended to OD_600_ = 0.6 in infiltration buffer [10 mM MgCl2, 10 mM MES (pH 5.6), 100 μM acetosyringone]. After 4 h of incubation at room temperature, equal volumes (1:1) of different combinations of the strain harboring each construct were mixed and co-infiltrated into tobacco leaves as described above.

The cell free proteasomal degradation assay was performed as described previously (García-Cano et al., 2014). For MG132 treatment, 10 μM MG132 or distilled water, was injected into tobacco leaves, respectively. After 4 h of injection, leaves were harvested and ground into fine powder in liquid nitrogen. Total protein extracts from 12.5 mg of fresh leaf weight, prepared by bead-beating the tissue in 25 μL of degradation/DNase digestion buffer [10 mM Tris-HCl (pH 7.6), 0.5 mM CaCl_2_, 10 mM MgCl_2_, 5 mM DTT, 5 mM ATP, and 1× plant protease inhibitor mixture (Sigma–Aldrich)], were incubated at room temperature for 30 min in a final reaction volume of 120 μl. The protein concentration was determined using the Bradford reagent (Bio-Rad Laboratories). Reactions were terminated by boiling in SDS sample buffer, and subjected to SDS-PAGE followed by western blotting using anti-GFP antibody (Abcam, Cat. No. ab290), as described above. Putative Rubisco large chains (∼55 kDa) was adopted as the loading control (Huang et al., 2013). LUC activity was also measured using the dual luciferase assay kit, as described above.

### Semi-*In Vivo* Analysis of MaICE1 Transactivational Activity

This transient expression assay was performed in tobacco leaves as described above. The *MaNAC1* promoter was cloned into the pGreenII 0800-LUC double reporter vector, while MaICE1 or MaSINA1, was cloned into the pEAQ vector as effectors (Primers are listed in Supplementary Table 1). The LUC and REN activity were recorded as described above. The *trans*-activation ability of MaICE1 to the *MaNAC1* promoter is indicated by the LUC/REN ratio. The experiments were repeated at least six times, yielding similar results.

## Results

### MaSINA1 Physically Interacts with MaICE1

**FIGURE 1:**
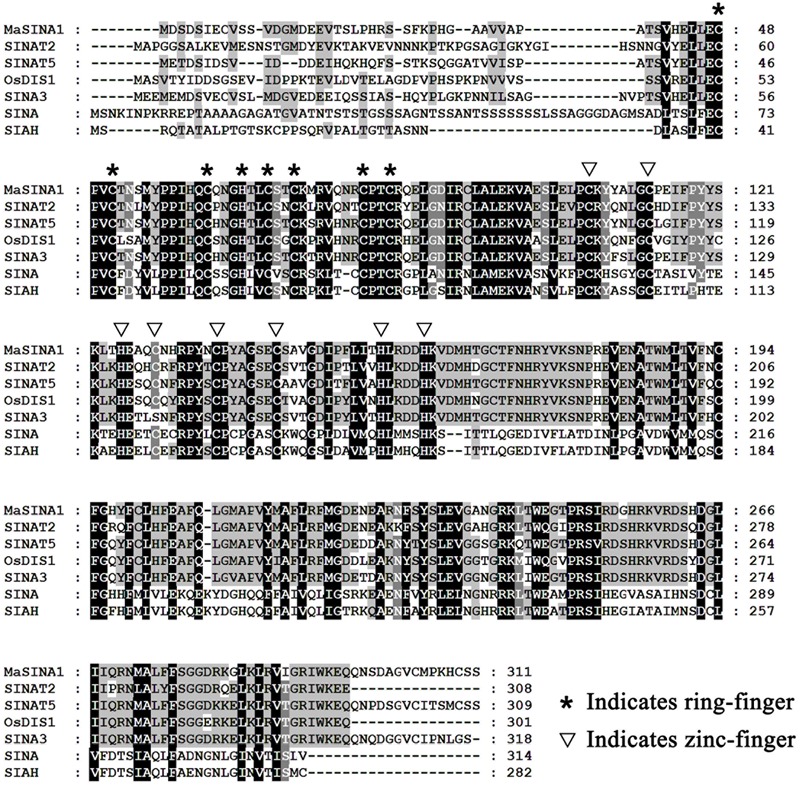
Multiple alignment of MaSINA1 and other SINA family proteins including *Arabidopsis* SINAT2 and SINAT5, rice OsDIS1, tomato SINA3, *Drosophila* SINA and human SIAH. The amino acid residues that are highly conserved among the examined proteins are shaded. The conserved ring-finger and zinc-finger motifs are indicated by asterisks and triangles, respectively.

**FIGURE 2:**
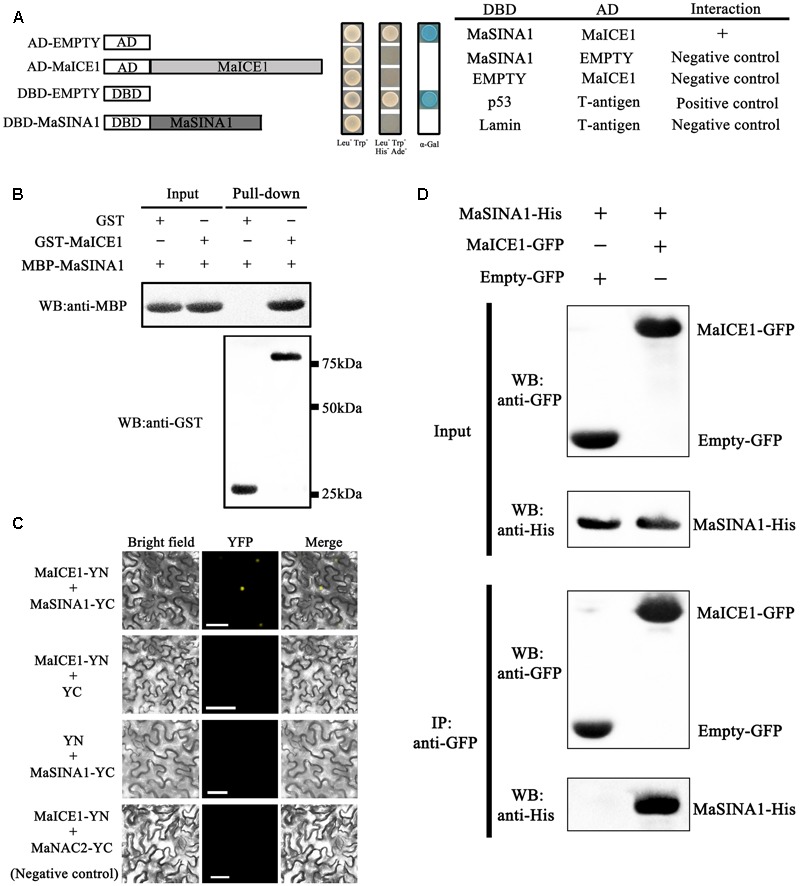
MaSINA1 interacts with MaICE1 *in vitro* and semi-*in vivo*. **(A)** Yeast two-hybrid assay for the interaction between MaSINA1 and MaICE1. The coding regions of MaSINA1 and MaICE1 were fused with DBD and AD vectors, respectively, as indicated, and co-transformed into the yeast strain Gold Y2H. The ability of yeast cells to grow on synthetic medium lacking tryptophan, leucine, histidine, and adenine but containing 125 μm Aureobasidin A, and to turn blue in the presence of the chromogenic substrate X-α-Gal, was scored as a positive interaction. **(B)** *In vitro* GST pull-down analysis of MaSINA1–MaICE1 interaction. MBP-MaSINA1 protein was incubated with GST-MaICE1 or GST, the bounded proteins were then detected by western blotting assays using the anti-His antibody and anti-GST antibody, respectively. **(C)** BiFC in tobacco leaf epidermal cells showing the interaction between MaSINA1 and MaICE1 in living cells. MaSINA1 fused with the C-terminus of YFP and MaICE1 fused with the N-terminus of YFP, were co-transfected into tobacco leaves and visualized using confocal microscopy. Expressions of MaSINA1 or MaICE1 alone, and MaICE1 with MaNAC2 were used as negative controls. YFP-fluorescence of YFP; Merge-digital merge of bright field and fluorescent images. The length of the bar indicated in the photographs is 30 μm. **(D)** CoIP assay showing the interaction between MaSINA1 with MaICE1. Tobacco leaves co-expressing MaICE1-GFP and MaSINA1-His, or empty-GFP and MaSINA1-His, was used to immunoprecipitate with the anti-GFP antibody, and the immunoblot was probed with the anti-GFP and anti-His antibody, respectively. These assays were conducted three biological repeats, yielding similar results.

In our previous study, ICE1–CBF pathway is acknowledged to be involved in cold tolerance of banana fruit (Peng et al., 2013; Zhao et al., 2013; Shan et al., 2014). In *Arabidopsis*, it has been well documented that ICE1 protein stability is vital for cold tolerance (Miura et al., 2011; Ding et al., 2015). To gain a deeper insight into regulators that will affect ICE1 stability of banana fruit, we screened a banana fruit cold stress-related cDNA library using MaICE1 as the bait. Among the eight candidate interacting proteins of MaICE1, one of the promising interactors (GSMUA_Achr9G05880_001; XP_009416451) was particularly interesting because it encodes a SEVEN IN ABSENTIA (SINA) ubiquitin ligase (termed MaSINA1), and it was studied further. The deduced protein of this gene contains conserved RING finger and zinc finger motifs, and has high sequence identity (85%) with *Arabidopsis* SINAT5 (Xie et al., 2002) (**Figure 1**), which was identified as a homolog of *Drosophila* SINA (Carthew and Rubin, 1990). The interaction between MaSINA1 and MaICE1 was further examined by yeast two-hybrid assay. As shown in **Figure 2A**, positive α-galactosidase activity confirmed the interaction between MaSINA1 and MaICE1. Then *in vitro* GST pull-down assay was performed to further confirm the interaction between MaSINA1 and MaICE1. The results showed that MBP-MaSINA1 protein was pulled down by GST-MaICE1 (**Figure 2B**), also supporting that MaSINA1 interacts with MaICE1.

Subsequently, we used BiFC to determine where MaSINA1-MaICE1 complex formation occurs within the plant cell. For this assay, MaSINA1 and MaICE1was fused with the C-terminal half and N-terminal half of the YFP, respectively. Both constructs were then transiently expressed in tobacco (*N. benthamiana*) leaves. Reconstituted YFP fluorescence was captured in the nucleus of the tobacco leave cells when MaSINA1-YC was coexpressed with MaICE1-YN, but not with the control combinations (**Figure 2C**), revealing that MaSINA1 interacts with MaICE1 in the nucleus.

To further validate this interaction in the plant cell, CoIP assay was performed in tobacco leaves expressing MaICE1-GFP and MaSINA1-His. The protein extraction was immunoprecipitated with the anti-GFP antibody, the immunoprecipitated protein complex was analyzed by western blotting using anti-GFP and anti-His antibodies. As shown in **Figure 2D**, the MaSINA1-His protein was only detected in the immunoprecipitated complex from the leaf tissue expressing MaSINA1-His and MaICE1-GFP. No MaSINA1-His protein was appeared in the immunoprecipitated complex from the leaf tissue expressing MaSINA1-His with the vector control (empty-GFP) (**Figure 2D**). These results together clearly demonstrate that MaSINA1 directly interacts with MaICE1.

### Molecular Characterization of MaSINA1

**FIGURE 3:**
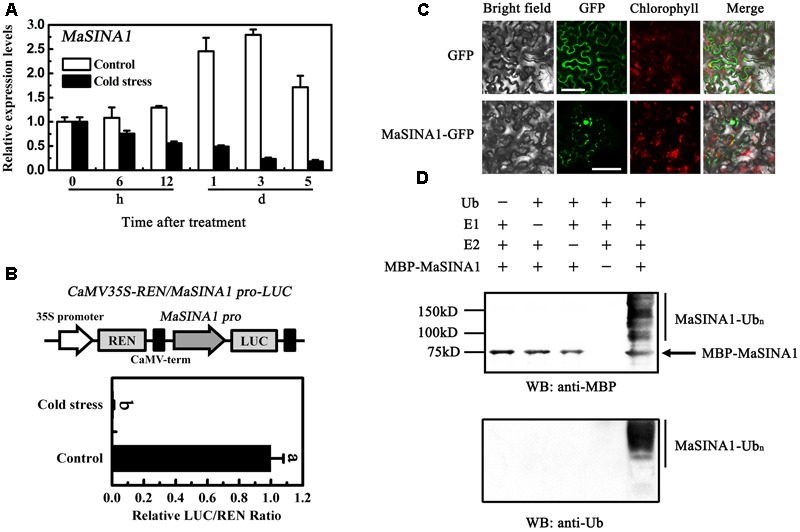
Molecular characterization of MaSINA1. **(A)** Expression of *MaSINA1* during cold storage. For cold stress, fruit were directly stored at 7°C, whereas for non-cold stress control, fruit were directly stored at 22°C. Expression level at different time points was expressed as a ratio relative to the harvest time (0 days of non-cold stress control), which was set at 1. Each value represents the means of three biological replicates, and vertical bars indicate the SE. **(B)** *MaSINA1* promoter activity in response to cold stress. The dual luciferase reporter vector containing *MaSINA1* promoter (CaMV35S–REN/*MaSINA1* pro-LUC) was transiently transformed into tobacco leaf protoplasts using a modified PEG method and test for cold stress (7°C) induction. After incubation for 36 h, LUC and REN luciferase activities were assayed, and the promoter activity is indicated by the ratio of LUC to REN. Each value represents the means of six biological replicates, and vertical bars represent the SE. Different letters above bars indicate significant difference at the 5% level by Student’s *t*-test. **(C)** Subcellular localization of MaSINA1 in tobacco leaves. MaSINA1 fused with the GFP or GFP positive control were infiltrated into tobacco leaves via *Agrobacterium tumefaciens* strain GV3101. After 48 h of the infiltration, GFP fluorescence was visualized using a fluorescence microscope. Red colors represent chlorophyll autofluorescent signals. Bars, 30 μm. **(D)** E3 ubiquitin ligase activity of MaSINA1. Recombinant MBP-MaSINA1 fusion protein was incubated in the presence or absence of E1, E2, and/or ubiquitin. The reactions were analyzed with immunoblots using anti-MBP and anti-ubiquitin antibodies. E3 ubiquitin ligase activity of MBP-MaSINA1 was only detected in the presence of E1, E2, and ubiquitin.

Unlike *MaICE1* expressed constitutively in the banana fruit under cold stress (Zhao et al., 2013), qRT-PCR analysis showed that *MaSINA1* was down-regulated by cold stress. Compared with the expression of *MaSINA1* in control fruit, the expression of *MaSINA1* in the fruit directly stored at 7°C (cold stress) decreased at 6 h, and was ∼14% of control on day 5 (**Figure 3A**).

To better understand the *MaSINA1* expression in response to cold stress, *MaSINA1* promoter with 1059 bp length was isolated from the genome of *M. acuminata*. Based on the Plant-CARE database, one low-temperature responsive element, CCGAC, termed LTRECOREATCOR15 was found in the *MaSINA1* promoter at -573 bp to -577 bp from the initiation codon (Supplementary Figure 1), indicating that *MaSINA1* promoter might be cold-responsive. Furthermore, a transient assay using the dual luciferase reporter system in tobacco leaf protoplasts showed that *MaSINA1* promoter activity was inhibited by cold stress (**Figure 3B**). These results indicate that *MaSINA1* is repressed by cold.

To investigate the subcellular location of MaSINA1, we fused the GFP with MaSINA1 protein, and transient expressed it in tobacco leaves. The GFP fluorescence of MaSINA1 fusion protein was localized predominantly in the nucleus, and some in the cytoplasm and plasma membrane (**Figure 3C**), which is similar with the localization of SINAT5 in *Arabidopsis* (Xie et al., 2002) and OsDIS1 in rice (Ning et al., 2011).

Previous studies showed that many RING motif-containing proteins possess E3 ubiquitin ligase activity (Stone et al., 2005). MaSINA1 contains a C3HC4-type RING finger motif at the N terminus with conserved Cys and His residues (**Figure 1**). To test whether MaSINA1 is a functional E3 ligase enzyme, we produced MaSINA1 in *E. coli* as a fusion with the MBP tag and purified the tagged protein (Supplementary Figure 2). Human E1, E2 and ubiquitin were used for the *in vitro* E3 ubiquitin ligase activity assay. Ubiquitination activity was detected using anti-MBP antibody and anti-ubiquitin antibody. As shown in **Figure 3D**, self-ubiquitination of MaSINA1 (polyubiquitinated smear ladders) was observed when Ub, E1, and E2 were present (**Figure 3D**, lane 5), but not in any negative controls that missed any one of the necessary components for the reaction (**Figure 3D**, lanes 1–4). Thus, MaSINA1 functions as an E3 ubiquitin ligase.

### MaSINA1 Targets MaICE1 for Ubiquitination Degradation

**FIGURE 4:**
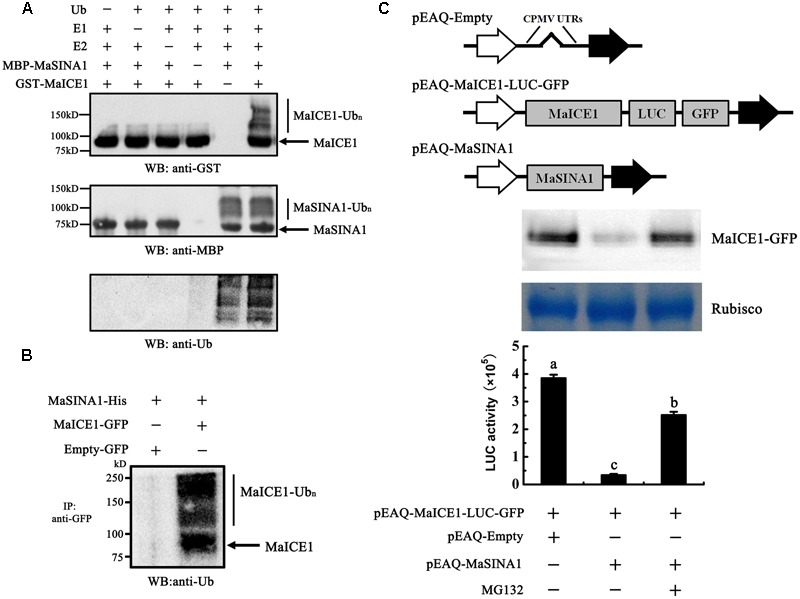
Ubiquitination of MaICE1 and degradation of MaICE1 in tobacco leaves by MaSINA1. **(A)** *In vitro* ubiquitination of MaICE1 by MaSINA1. Recombinant MBP-MaSINA1 protein was co-incubated with GST-MaICE1 protein in the presence or absence of Ub, human E1, and human E2 at 30°C for 2 h. The reaction mixture was analyzed by immunoblotting with the anti-GST, anti-MBP, and anti-ubiquitin antibody, respectively. Ubiquitination results in a heterogeneous collection of higher-molecular mass proteins that were detected using these antibodies. **(B)** Semi-*in vivo* ubiquitination of MaICE1 by MaSINA1. Tobacco leaves co-expressing MaICE1-GFP and MaSINA1-His, or empty-GFP and MaSINA1-His, was used to immunoprecipitate with the anti-GFP antibody, and the immunoblot was probed with the anti-ubiquitin antibody. **(C)** Proteasome-mediated degradation assay of MaICE1 in plant cells. Up panel, Western blot analysis of MaICE1 degradation in the tobacco cell-free system. As indicated, MaICE1 fused with GFP and LUC was expressed alone or co-expressed with MaSINA1 in tobacco leaves in the presence or absence of MG132. The resulting protein extracts were analyzed using an anti-GFP antibody. Putative Rubisco large chains (∼55 kDa) was adopted as the loading control for total protein. Bottom panel, LUC activity in each sample used in Western blot analysis. Each value represents the mean ± SE of six biological replicates. Different letters above bars indicate a statistical difference at the 5% level compared with the empty.

As MaSINA1 interacts with MaICE1 (**Figure 1**), and MaSINA1 functions as an E3 ubiquitin ligase, we sought to investigate whether MaICE1 protein could be ubiquitinated by MaSINA1, an *in vitro* ubiquitination assay was also carried out using MaICE1 protein as a substrate. Recombinant GST-MaICE1 and MBP-MaSINA1 proteins were co-incubated in the presence or absence of Ub, E1, and E2 at 30°C for 2 h. The reaction mixture was analyzed by immunoblotting using anti-GST antibody. Co-incubation of GST-MaICE1 and MBP-MaSINA1 in the presence of Ub, E1, and E2 gave rise to a high-molecular-mass band, while lacking of Ub, E1, or E2 in the reaction mixture abolished the ubiquitinated band (**Figure 4A**), suggesting that MaSINA1 is a ubiquitin ligase capable of ubiquitinating MaICE1. The ubiquitination of MaICE1 by MaSINA1 was further confirmed *in planta*. MaICE1-GFP and MaSINA1-His constructs were co-expressed in *N. benthamiana* leaves. After immunoprecipitation with the anti-GFP antibody matrix, the immunoprecipitated complex was verified by Western blotting using the anti-ubiquitin antibody. As shown in **Figure 4B**, a smear banding representing the polyubiquitinated MaICE1 protein was detected by the anti-ubiquitin antibody in the anti-GFP-immunoprecipitated complex from the co-expression of MaICE1-GFP and MaSINA1-His, but not in the immunoprecipitated complex from the co-expression of empty-GFP and MaSINA1-His control (**Figure 4B**). Together, these data reveal that MaSINA1 is able to ubiquitinate MaICE1.

To test whether MaSINA1 can promote MaICE1 degradation through ubiquitination, the stability of MaICE1 protein in plant cells was next examined using a cell free proteasome degradation assay. The *MaICE1* fused to a GFP and a LUC tag was inserted into pEAQ vector (**Figure 4C**). Total proteins were extracted from tobacco leaves transiently co-expressing GFP- and LUC-tagged MaICE1 and/or MaSINA1, and the stability of MaICE1-GFP was tested by western blotting with anti-GFP antibody. As shown in **Figure 4C**, MaICE1 amounts declined substantially in the presence of MaSINA1, whereas without MaSINA1, MaICE1 remained relatively stable. This MaSINA1-mediated destabilization of MaICE1 most likely occurred by proteasome degradation pathway, because it was inhibited by MG132, a known selective inhibitor of proteasomal activity (Yang et al., 2004). Quantification of LUC activity also showed that expression of MaSINA1 protein resulted in the degradation of MaICE1 protein, while in the presence of MG132, the degradation of MaICE1 was eliminated (**Figure 4C**). Together, these findings strongly demonstrate that MaSINA1 and MaICE1 form a protein module by which MaSINA1 ubiquitinates and controls the steady protein level of MaICE1.

### MaSINA1 Attenuates the *Trans*-activation of MaICE1 to MaNAC1

**FIGURE 5:**
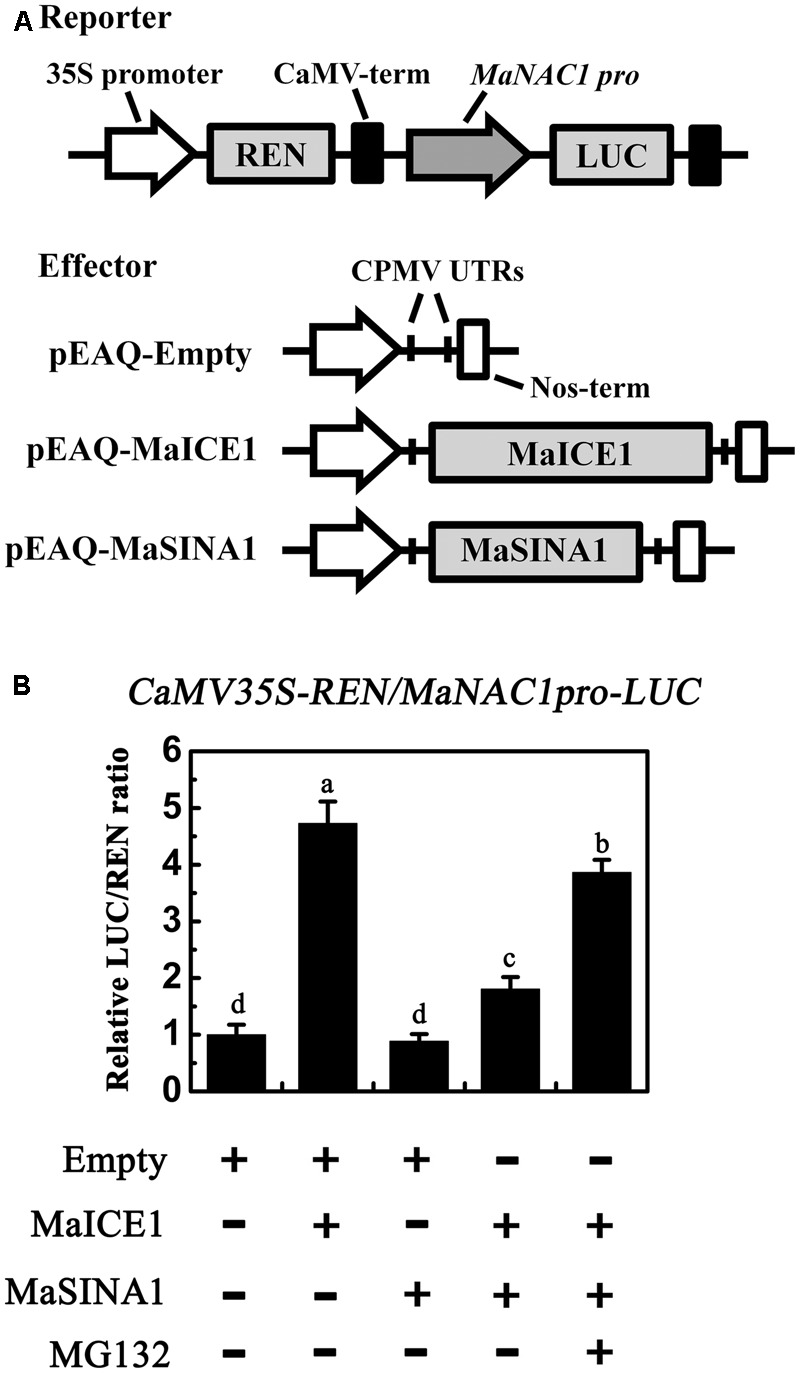
The *trans*-activation of MaICE1 to *MaNAC1* promoter is attenuated by MaSINA1, and the attenuation is diminished by the proteasome inhibitor MG132. **(A)** The reporter and effector vectors used in the transient assay. **(B)** *Agrobacterium* strain GV3101 carrying the reporter plasmid and different combinations of effector plasmids was infiltrated into tobacco leaves, and the luciferase activity at the site of infiltration was measured 3 days after infiltration. The activities of LUC and REN were measured sequentially, and the LUC/REN ratio was calculated as the final transcriptional activation activity. Data represent the mean ± SE of six biological replicates. Different letters above bars indicate a statistical difference at the 5% level between combinations by Student’s *t*-test.

Our previous study showed that MaICE1 bound specifically to the MYC recognition sequence in the *MaNAC1* promoter and positively regulated *MaNAC1* expression, which is an important transcriptional regulator of cold stress response of banana fruit (Shan et al., 2014). The result that MaSINA1 targets MaICE1 for ubiquitination degradation led us to investigate whether or not MaSINA1 interferes with *trans*-activation of *MaNAC1* by MaICE1. To this end, we performed transient expression assays using the dual-luciferase reporter system. In this experiment, the *MaNAC1* promoter-driven LUC (*MaNAC1* pro-LUC) and CaMV35S promoter-driven REN (CaMV35S-REN; as an internal control) were constructed in the same vector, together with an effector plasmid expressing MaICE1 or MaSINA1 (**Figure 5A**), and expressed in the tobacco leaves. The *in vivo* transcriptional activity of MaICE1 was reflected by the LUC/REN ratio. Compared with the control that was co-transfected with the empty construct, co-expression of MaICE1 with *MaNAC1 Pro*-LUC significantly increased the LUC/REN ratio, while this increase was abolished when MaSINA1 was co-expressed (**Figure 5B**), indicating that MaSINA1 attenuates the *trans*-activation of MaICE1 to *MaNAC1*. Importantly, in the presence of MG132, the attenuation of MaSINA1 on MaICE1 transcriptional activation of *MaNAC1* was diminished (**Figure 5B**). These data indicate that MaSINA1 might act as a negative regulator of cold stress response of banana fruit via controlling the stability and *trans*-activation of MaICE1.

## Discussion

Cold storage is effectively applied to maintain the post-harvest qualities and extend the shelf life of many horticultural fruits and vegetables. However, tropical and subtropical fruits, like bananas, are easily susceptible to chilling injury, resulting in quality deterioration and substantial losses, which significantly shortens storage life (Chen et al., 2008; Zhao et al., 2013; Shan et al., 2014). Thus, revealing the molecular mechanism(s) of the cold response in banana fruit is important for the genetic improvement of cold tolerance and improving fruit quality and storage potential. Currently, the most well-known cold signaling pathway is ICE-CBF-COR cascade (Ding et al., 2015; Shi et al., 2015). Accordingly, we also previously found that ICE-CBF pathway is positively involved in cold stress response of banana fruit (Zhao et al., 2013; Shan et al., 2014). However, regulators that act upstream in this cascade have not been identified. In this study, we sought to identify a SINA ubiquitin ligase MaSINA1 from banana fruit, and show that MaSINA1 controls MaICE1 protein stability via the UPS.

The UPS serves as a versatile post-translational modification, and has been implicated in almost all aspects of growth and development, as well as in responses to biotic and abiotic stress in plants (Lyzenga and Stone, 2012; Stone, 2014; Sharma et al., 2016). Specificity of the UPS is controlled mainly by the substrate-recruiting E3 ubiquitin ligases, and consequently, a large number of E3 ligases, have been isolated and shown to be involved in plant stress responses by modulating the abundance of key downstream stress-responsive TFs (Lyzenga and Stone, 2012; Stone, 2014; Sharma et al., 2016). However, only a few ubiquitin ligases such as HOS1, CONSTITUTIVE PHOTOMORPHOGENIC 1 (COP1), carboxyl terminus of Hsc70-interacting protein (CHIP) and *Arabidopsis* F-box protein 7 (FBP7), have been implicated in cold stress response (Barrero-Gil and Salinas, 2013). *Arabidopsis* HOS1 was the first RING-type E3 ubiquitin ligase to act as a negative regulator of cold responses (Jung et al., 2014). Under normal growth conditions, HOS1 did not affect the nuclear localization and the abundance of GFP-ICE1, while HOS1 can interact with and ubiquitinate ICE1 for degradation under cold stress (Dong et al., 2006). Consistent with a role in mediating ICE1 degradation, overexpression of HOS1 results in reduced expression of cold- responsive genes and increased sensitivity to cold stress (Lee et al., 2001), suggesting HOS1 functions to attenuate stress signaling. Strangely, no interaction was found between banana fruit MaHOS1 and MaICE1 (data not shown). Indeed in the present work, another RING-type E3 termed MaSINA1 was identified from banana fruit. MaSINA1 showed high sequence similarity to *Arabidopsis* SINAT5 (**Figure 1**), and was repressed by cold stress (**Figure 3**). We also found that MaSINA1 interacted with and ubiquitinated MaICE1 and promoted MaICE1 degradation via the UPS (**Figures 2, 4**). In addition, we demonstrated that MaSINA1 attenuated the *trans*-activation of MaICE1 to MaNAC1 (**Figure 5**). Based on these results, it could be speculated that MaSINA1 negatively regulates cold stress response of banana fruit by promoting the ubiquitination-mediated degradation of MaICE1 protein. To the best of our knowledge, it is the first report that except HOS1, another novel E3 ubiquitin ligase controls ICE1 protein stability in fruits. Previously, we also showed that unlike *Arabidopsis* ICE1, cold-induced phosphorylation of MaICE1 might not be necessary for its transcriptional activity, but could enhance its *trans*-activation ability (Shan et al., 2014). The present data also reveal that the regulators of controlling ICE1 stability via UPS during cold stress response might be different in banana fruit and *Arabidopsis*. However, whether MaICE1 stability controlled by MaSINA1 is cold-dependent or -independent needs to be elucidated. In addition, it should be pointed out that ubiquitination is a reversible post-translational modification and de-ubiquitination enzymes (ubiquitin proteases) are also involved in modulating protein function (Barrero-Gil and Salinas, 2013). Therefore, the participation of ubiquitin proteases in plant responses to cold stress will be a subject of future research.

Besides ubiquitination, protein phosphorylation and sumoylation also play an important role in the regulation of ICE1 activity under cold stress (Chinnusamy et al., 2007; Barrero-Gil and Salinas, 2013). In contrast with HOS1, SIZ1 mediates sumoylation of ICE1, which reduces the polyubiquitination of ICE1 to enhance its stability (Miura et al., 2007). Although a very earlier research hypothesized that cold stress induces phosphorylation of ICE1 (Chinnusamy et al., 2007), until recently, the hypothesis is confirmed by Ding et al. (2015). They found that under cold stress, OST1 (OPEN STOMATA 1), a well-known Ser/Thr protein kinase in ABA signaling, phosphorylates ICE1 to antagonize the degradation of ICE1 mediated by HOS1. More interestingly, the OST1 protein also competes with HOS1 to bind to ICE1, thus releasing ICE1 from the HOS1-ICE1 complex. Thus, the dual role of OST1 contributes to the enhancement of ICE1 stability (Ding et al., 2015). Our previous study also indicated that the *trans*-activation ability of MaICE1 is enhanced by MaICE1 phosphorylation. Given that ICE1 protein stability is regulated by various post-translational modifications, it is of interest to find out how these modifications are coordinately balanced to maintain ICE1 homeostasis in response to cold stress.

## Conclusion

In summary, a banana fruit SINA ubiquitin ligase MaSINA1 was identified. MaSINA1 can ubiquitinate MaICE1 for the 26S proteasome-dependent degradation, and therefore suppresses the transcriptional activation of MaICE1. Our findings reveal a novel mechanism in banana fruit for control of the stability of ICE1 and for the negative regulation of cold stress response by a SINA E3 ligase.

## Author Contributions

J-YC, W-JL, and WS designed the research. Z-QF, and WS performed the experiments. Z-QF, J-YC, J-FK, W-JL, and WS wrote the manuscript.

## Conflict of Interest Statement

The authors declare that the research was conducted in the absence of any commercial or financial relationships that could be construed as a potential conflict of interest.

## Footnotes

1 http://bioinformatics.psb.ugent.be/webtools/plantcare/html/
