# Supplementary material for: The Banana Fruit SINA Ubiquitin Ligase MaSINA1 Regulates the Stability of MaICE1 to be Negatively Involved in Cold Stress Response
Source: Front Plant Sci. 2017 Jun 12;8:995. doi: 10.3389/fpls.2017.00995 (PMC5467002; doi:10.3389/fpls.2017.00995)
Supplement: Supplementary file 1 [file Table_1.DOC]

**Supplementary Table 1** Summary of primers used in this study.

| **Assay** | **Primer sequence** | **Restriction Site** |
| --- | --- | --- |
| **RT-qPCR** | ***MaSINA1*-F: *CGACCGTATAATTGCCCATAT***  ***MaSINA1*-R: *TTGCTTCATTCTCGTCACCC*** |  |
| **Promoter isolation** | ***MaSINA1PRO*-F: AGGTTGACGCGAGTTAGCTGTCT**  ***MaSINA1PRO*-R: GCCTCCAGCGGTCAGGGATTCAA** |  |
| **Subcellular localization** | ***MaSINA1-pEAQGFP-F:* CCCAAATTCGCG*accggt*ATGGACTCCGACAGCATCGAGTG**  ***MaSINA1-pEAQGFP-R:* TTCTCCTTTGCTAGTCATGCTGCTGCAGTGTTTTGGCATG** | ***Age* I** |
| **Ubiquitination assays** | ***MaSINA1-pMAL-F:* GGAAGGATTTCA*gaattc*ATGGACTCCGACAGCATCGAGT**  ***MaSINA1-pMAL-R:* CTGCAGGTCGAC*tctaga*TCAGCTGCTGCAGTGTTTTGGC**  ***MaICE1-pGEX-F:* CGTGGATCCCCG*gaattc*ATGCTGGACGACGACGACGACA**  ***MaICE1-pGEX-R:* ACGATGCGGCCG*ctcgag*TCATGACACTGTATTATCGAAGCCG**  ***FLUC-GFPpEAQ-F*: CCCAAATTCGCG*accggt*ATGGAAGACGCCAAAAACATAA**  ***FLUC-GFPpEAQ-R*:TTCTTCTCCTTTGCTAGTCATCACGGCGATCTTTCCGCCCTTC**  ***MaICE1-FLUC-GFPpEAQ-F:*CCCAAATTCGCG*accggt*ATGCTGGACGACGACGACGACA**  ***MaICE1- FLUC-GFPpEAQ-R:*ATGTTTTTGGCGTCTTCCATTGACACTGTATTATCGAAGCC**  ***MaSINA1-pEAQ-F:* CCCAAATTCGCG*accggt*ATGGACTCCGACAGCATCGAGT**  ***MaSINA1-pEAQ-R:* AGAGTTAAAGGC*ctcgag*GCTGCTGCAGTGTTTTGGCATG** | ***Eco*R I**  ***Xba* I**  ***Eco*R I**  ***Xho* I**  ***Age* I**  ***Age* I**  ***Age* I**  ***Age* I**  ***Age* I**  ***Xho* I** |
| **CoIP** | ***MaSINA1-pEAQHis-F:* CCCAAATTCGCG*accggt*ATGGACTCCGACAGCATCGAGT**  ***MaSINA1-pEAQHis-R:* TGATGGTGATGGTGATG*cccggg*GCTGCTGCAGTGTTTTGGCATG**  ***MaICE1-pEAQGFP-F:* CCCAAATTCGCG*accggt*ATGCTGGACGACGACGACGACA**  ***MaICE1-pEAQGFP-R:* TTCTCCTTTGCTAGTCATTGACACTGTATTATCGAAGCCG** | ***Age* I**  ***Sma* I**  ***Age* I**  ***Age* Z** |
| **Dual-luciferase transient**  **expression assay** | ***MaICE1-pEAQ-F:* CCCAAATTCGCG*accggt*ATGCTGGACGACGACGACGACA**  ***MaICE1-pEAQ-R:* AGAGTTAAAGGC*ctcgag*TGACACTGTATTATCGAAGCCG**  ***MaSINA1PRO-LUCReporter-F:* TATAGGGCGAATTG*ggtacc*AGGTTGACGCGAGTTAGCTGTCT**  ***MaSINA1PRO- LUCReporter-R:* ATGTTTTTGGCGTCTTCCATGCCTCCAGCGGTCAGGGATTCAA** | ***Age* I**  ***Xho* I**  ***Kpn* I**  ***Nco* I** |
| **Yeast two-hybrid** | ***MaICE1-AD-F:* CCATGGAGGCCAGT*gaattc*ATGCTGGACGACGACGACGACA**  ***MaICE1-AD-R:* GCTCGAGCTCGAT*ggatcc*CTGACACTGTATTATCGAAGCCG**  ***MaSINA1-BD-F:* GGCCATGGAGGCC*gaattc*ATGGACTCCGACAGCATCGAGT**  ***MaSINA1-BD-R:* CGGCCGCTGCAG*gtcgac*GGCTGCTGCAGTGTTTTGGCATG** | ***EcoR* I**  ***BamH* I**  ***EcoR* I**  ***Sal* I** |
| **BiFC** | ***MaICE1-YNE-F:* ATATTCTGCCCAAATTCGCGATGCTGGACGACGACGACGACA**  ***MaICE1-YNE-R:* CCCTTGCTCACCAT*accggt*TGACACTGTATTATCGAAGCCG**  ***MaSINA1-YCE- F:* GCATGGACGAGCTGTACAAG*ctcgag*ATGGACTCCGACAGCATCGAGT**  ***MaSINA1-YCE- R:* TTAATGAAACCAGAGTTAA*aggcct*TCAGCTGCTGCAGTGTTTTGGC** | ***Age* I**  ***Age* I**  ***Xho* I**  ***Stu* I** |
