# Supplementary material for: The Banana Fruit SINA Ubiquitin Ligase MaSINA1 Regulates the Stability of MaICE1 to be Negatively Involved in Cold Stress Response
Source: Front Plant Sci. 2017 Jun 12;8:995. doi: 10.3389/fpls.2017.00995 (PMC5467002; doi:10.3389/fpls.2017.00995)
Supplement: Supplementary file 2 [file Image_1.PDF]

GTTGACGCGAGTTAGCTGTCTATTTTGTATGGTGTCGTTGCGTATGCTATCGGATAAAGCAATATTAG
AACAATGTGATGGTGTGTTTGCATCCTGATTTGTTACTATTACGGTTCATGCATTGATCCTATGAAGC
AATATTAGAACAATGTGGTGGTATGTTTGCATTCTGATTTGTTACTATTACGGTTCATGCTTTGGTCCT
ATGAAGCAATATCAGAACAATATGGTGGTATGATTGCATTCTGATTTGTTACTATTACGGTTCATGCA
TTGATCCTATGAAGCAGTATTAGAACAATGTGGTGGTATGATTGCATGCTGATTTGTTACTATTACTG
TTCATGCGTTGATGCTGGAGCGAGGTAAATGACGAAACTGGGACTCAGCCAGGTGTCTTGTATGAAG
AATAGGTCAACATTTTGTGGTGTTCTCCTGTTTATTTTTGTACTCTGATGTACCCAATATAGAGGCCTG
TGGA**<u>CCGAC</u>**CAACACGTGGATGGCCGTTCAAAGGATTCAAACACTAAACTGAAAAAAGAAAATCCT
TTTTTATTGGTAAAAAGAAAACGGAACTTTGCACATCCGAAATTTGCGTCACAAGATTTTAATTATGA
ATAAAAAATTCAAAATTTATTAGGCAAACAAAATAAATAAATCAAGTAGTAAATCGTTATATGGTCG
AACGTCCGTTCCCAATCAACCTTTCTTTTCCCGTTAATTTCTATGTAGGCTGACGACCTTGGATCAAGT
TAACCGGGCCCCACAGGGCTGCCTCCAATCACAGCGCCGGTGGGTGACTGGGCGTCATGACAAAAA
ATAAATATAAAGGACCCGAAAAGAATTGCGGTGATACCGTCACCTCCTGTTAGTGCGGGCCGAGACC
TCCTCCTCCTCACACCTCGCTTTGGTTCCTAGCCCTCCTCTCTCTGTCTTCTGCGTCATAATCTCTTCAA
AGGCGCTCCCTTTCCTTTGGTCGCCACCCTTTCCAGGCTGATTTTGGAGAGCGGAGAGGGCGACGGG
CGGGGAGGGCACGAGATCCTTGAATCCCTGACCGCTGGAGGCATG

Supplementary Figure 1. The promoter nucleotide sequence of MaSINA1. The low-temperature responsive element (CCGAC) was underlined.
