# Supplementary material for: The Banana Fruit SINA Ubiquitin Ligase MaSINA1 Regulates the Stability of MaICE1 to be Negatively Involved in Cold Stress Response
Source: Front Plant Sci. 2017 Jun 12;8:995. doi: 10.3389/fpls.2017.00995 (PMC5467002; doi:10.3389/fpls.2017.00995)
Supplement: Supplementary file 3 [file Image_2.PDF]

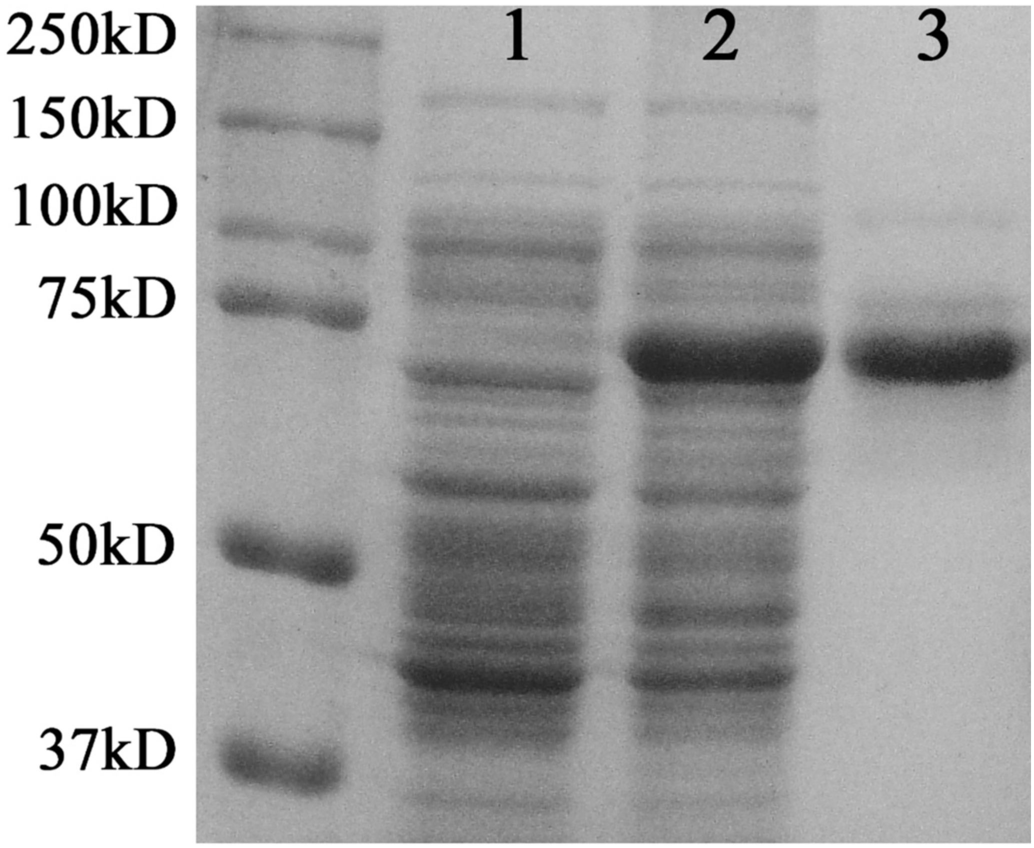

Supplementary Figure 2. SDS-PAGE gel stained with Coomassie blue demonstrating affinity purification of the recombinant MaSINA1 protein used for in vitro ubiquitination assay. Lane 1, Non-induced protein; Lane 2, Before purification; Lane 3, After purification.
